# P1 of turnip mosaic virus interacts with NOD19 for vigorous infection

**DOI:** 10.3389/fmicb.2023.1216950

**Published:** 2023-06-23

**Authors:** Esther O. Bello, Yingshuai Yang, Yue Fang, Mengzhu Chai, Xue Jiang, Yameng Luan, Yuting Wang, Yating Guo, Xiao-Yun Wu, Xiaofei Cheng, Xiao-Xia Wu

**Affiliations:** ^1^Key Laboratory of Germplasm Enhancement, Physiology and Ecology of Food Crops in Cold Region of the Ministry of Education, Northeast Agricultural University, Harbin, Heilongjiang, China; ^2^College of Agriculture, Northeast Agricultural University, Harbin, Heilongjiang, China

**Keywords:** *Potyvirus*, turnip mosaic virus, soybean mosaic virus, P1, NOD19, infection

## Abstract

P1 protein, the most divergent protein of virus members in the genus *Potyvirus* of the family *Potyviridae*, is required for robust infection and host adaptation. However, how P1 affects viral proliferation is still largely elusive. In this work, a total number of eight potential P1-interacting Arabidopsis proteins were identified by the yeast-two-hybrid screening using the turnip mosaic virus (TuMV)-encoded P1 protein as the bait. Among which, the stress upregulated NODULIN 19 (NOD19) was selected for further characterization. The bimolecular fluorescent complementation assay confirmed the interaction between TuMV P1 and NOD19. Expression profile, structure, and subcellular localization analyses showed that NOD19 is a membrane-associated protein expressed mainly in plant aerial parts. Viral infectivity assay showed that the infection of turnip mosaic virus and soybean mosaic virus was attenuated in the null mutant of Arabidopsis NOD19 and NOD19-knockdown soybean seedlings, respectively. Together, these data indicate that NOD19 is a P1-interacting host factor required for robust infection.

## 1. Introduction

The genus *Potyvirus* in the family *Potyviridae* contains numerous widespread and economically important plant viruses that infect a wide range of crops, including vegetables, fruits, and field crops (Moury and Desbiez, 2020). Potyviruses have long, flexuous filamentous virions that are primarily spread by aphids in nature but can also be transmitted through seed and occasionally by direct contact (Coutts and Jones, 2015). Potyviruses can cause significant economic losses by reducing yields and market value of infected crops (Yang et al., 2021). The genome of potyviruses is composed of a single-stranded positive-sense RNA that encodes only one open reading frame (ORF). The ORF is translated into a large polyprotein that is cleaved into 10 mature proteins by three viral proteases which are P1 PROTEIN (P1), HELPER COMPONENT PROTEINASE (HcPro), P3 PROTEIN (P3), 6-KILODALTON PROTEIN 1 (6 K1), CYLINDRICAL INCLUSION (CI), 6-KILODALTON PROTEIN 2 (6 K2), VIRAL PROTEIN GENOME-LINKED (VPg), NUCLEAR INCLUSION A-PROTEINASE (NIa-Pro), NUCLEAR INCLUSION B (NIb), and COAT PROTEIN (CP; Inoue-Nagata et al., 2022). In addition, all potyviruses contains a polymerase slippage motif within the *P3* cistron (GAAAAAA), which allow the expression of an additional short polyprotein that is cleaved into P1, HcPro, and P3 N-TERMINAL FUSED WITH PRETTY INTERESTING POTYVIRIDAE ORF (P3N-PIPO; Chung et al., 2008). Noticeably, some sweet potato-infecting potyviruses also contain a polymerase slippage motif within the P1 cistron, which allow the expression of another protein named P1 N-TERMINAL FUSED WITH PRETTY INTERESTING SWEETPOTATO POTYVIRUS ORF (P1N-PISPO; Mingot et al., 2016; Untiveros et al., 2016).

P1 protein is located at the N-terminus of the potyviral polyprotein and is the most divergent of all potyviral proteins (Rohozkova and Navratil, 2011). Despite this variability, the C-terminal regions of potyviral P1 harbors a well conserved serine protease domain that is responsible for the *cis*-cleavage of the P1-HcPro junction and subsequent release of P1 from the polyprotein (Verchot et al., 1991). The N-terminal part of P1 is highly variable in both size and sequence (Verchot et al., 1992; Shan et al., 2018). Interestingly, the protease activity of the serine protease domain is tightly regulated by its N-terminal variable part at the assistance of an unknown host factor (Verchot et al., 1992). Moreover, changing the N-terminal sequence of potyviruses always results in the alteration of their host range and/or pathogenicity (Salvador et al., 2008; Mao et al., 2022). Although P1 is not strictly required for potyviral genome amplification and movement but the separation of P1 and HcPro is essential to stimulate the RNA silencing suppressing activity of HcPro and viral viability (Verchot and Carrington, 1995; Tena Fernández et al., 2013). P1 also functions as an accessory factor for robust genome amplification possible by suppressing host antiviral defenses (Pasin et al., 2014, 2020). P1 is a nucleic acid binding protein that locates both in the cytoplasm and nucleus (Soumounou and Laliberté, 1994; Arbatova et al., 1998; Martínez and Daròs, 2014). Despite the important roles of P1 in viral proliferation, only a few P1-interacting host factors have been identified, namely the CYTOCHROME B6/F COMPLEX RIESKE IRON-SULFUR PROTEIN (Rieske Fe/S), CHLOROPLAST SIGNAL RECOGNITION PARTICLE 54 (cpSRP54), PURPLE ACID PHOSPHATASE 2.1 (PAP2.1), HEAT-SHOCK PROTEIN 70 (HSP70), TUDOR-STAPHYLOCOCCAL NUCLEASE (Tudor-SN), and ribosomal subunits (Shi et al., 2007; Martínez and Daròs, 2014; Ji et al., 2021; Widyasari et al., 2022). In this study, eight additional P1-interacting candidates were identified via a yeast-two-hybrid (Y2H) screening and the roles of one host factor, namely stress upregulated NODULIN 19 (NOD19), in viral proliferation was described.

## 2. Results

### 2.1. Identification of P1-interacting factors by Y2H screen

**Figure 1:**
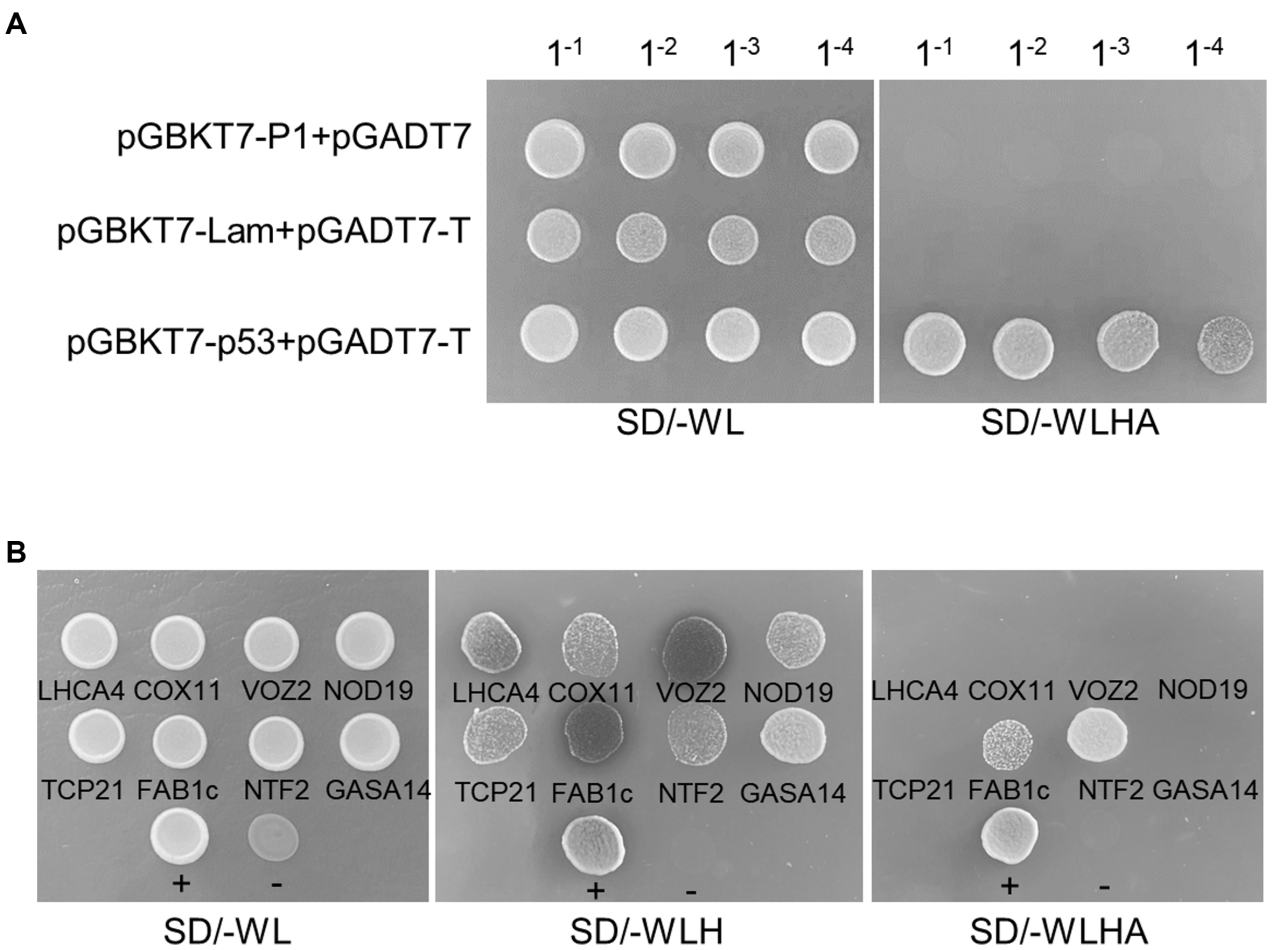
Y2H Screen of P1-interacting host factors. **(A)** Growth of serially diluted yeast cells that were transformed with the indicated plasmids on selective media. Lam, the human lamin C; p53, the tumor suppressor p53; T, SV40 large T-antigen. **(B)** Growth of yeast cells transformed with pGBKT7-P1 and the fragments of eight P1-interacting candidates in pGADT7 vector on −WL, −WLH, or −WLHA media. +, positive control (pGBKT7-p53+pGADT7-T); -, negative control (pGBKT7-Lam+pGADT7-T).

To identify P1-interacting host factors, we performed a Y2H screen of *Arabidopsis thaliana* cDNA library using turnip mosaic virus (TuMV)-encoded P1 as the bait. Yeast strain AH109 co-transformed with pGADT7 empty vector and pGBK-P1 did not survive on the selective media lacking tryptophan, leucine, histidine, and adenine (−WLHA), indicating that TuMV P1 has no self-activation activity (Figure 1A). The bait plasmid was then cotransformed with *A. thaliana* cDNA library in the pGADT7 vector. To avoid missing weak P1-interacting host factors, two rounds of screen were performed on the −WLHA and −WLH selective media, respectively. A total number of five and 18 colonies were recovered on the −WLHA and −WLH selective media, respectively. Plasmids were extracted from each of these colonies and Sanger sequenced. The results showed that the insertions of the 23 plasmids belong to eight genes: stress upregulated NOD19, VASCULAR ONE ZINC FINGER PROTEIN 2 (VOZ2), TEOSINTE BRANCHED1/CYCLOIDEA/PROLIFERATING CELL FACTOR 21 (TCP21), FORMS APLOID AND BINUCLEATE CELLS 1C (FAB1c), CYTOCHROME C OXIDASE 11 (COX11), NUCLEAR TRANSPORT FACTOR 2 (NTF2), LIGHT-HARVESTING CHLOROPHYLL-PROTEIN COMPLEX I SUBUNIT A4 (LHCA4), and the GIBBERELLIC ACID (GA)-STIMULATED IN ARABIDOPSIS 14 (GASA14; Table 1). We retransformed yeast cells with pGBKT7-P1 and each of the eight genes in pGADT7 vector and plated on −WLH or −WLHA selective media. The results showed that yeast cells transformed with all combinations of plasmids survived on −WLH media, but only those transformed with pGBKT7-P1 and either pGADT7-FAB1c or pGADT7-NTF2 grew on −WLHA media (Figure 1B), indicating that P1 may interact strongly with FAB1c and NTF2 and weakly with the other six proteins. In this study, we report the interaction between P1 and NOD19 and the biological function of the interaction in viral proliferation. The biological function of the interaction between P1 and other genes will be reported elsewhere.

**Table 1:** The putative P1-interacting host factors obtained in the Y2H assay.

No. of clones	Gene name	Description
8	TCP21	TCP family transcription factor 11 (At5g08330)
4	FAB1c	Forms aploid and binucleate cells 1C (FAB1C; At1g71010)
3	NOD19	Stress upregulated NODULIN 19 (AT5G61820)
3	VOZ2	Vascular one zinc finger protein 2 (At2g42400)
2	COX11	Cytochrome C oxidase 11 (AT1G02410)
1	NTF2	Nuclear transport factor 2 (NTF2; AT5G60980)
1	LHCA4	Light-harvesting chlorophyll-protein complex I subunit A4 (AT3G47470)
1	GASA14	Alpha/beta-hydrolases superfamily protein (AT5G14390)

### 2.2. TuMV P1 interacts with NOD19 outside the nucleus

**Figure 2:**
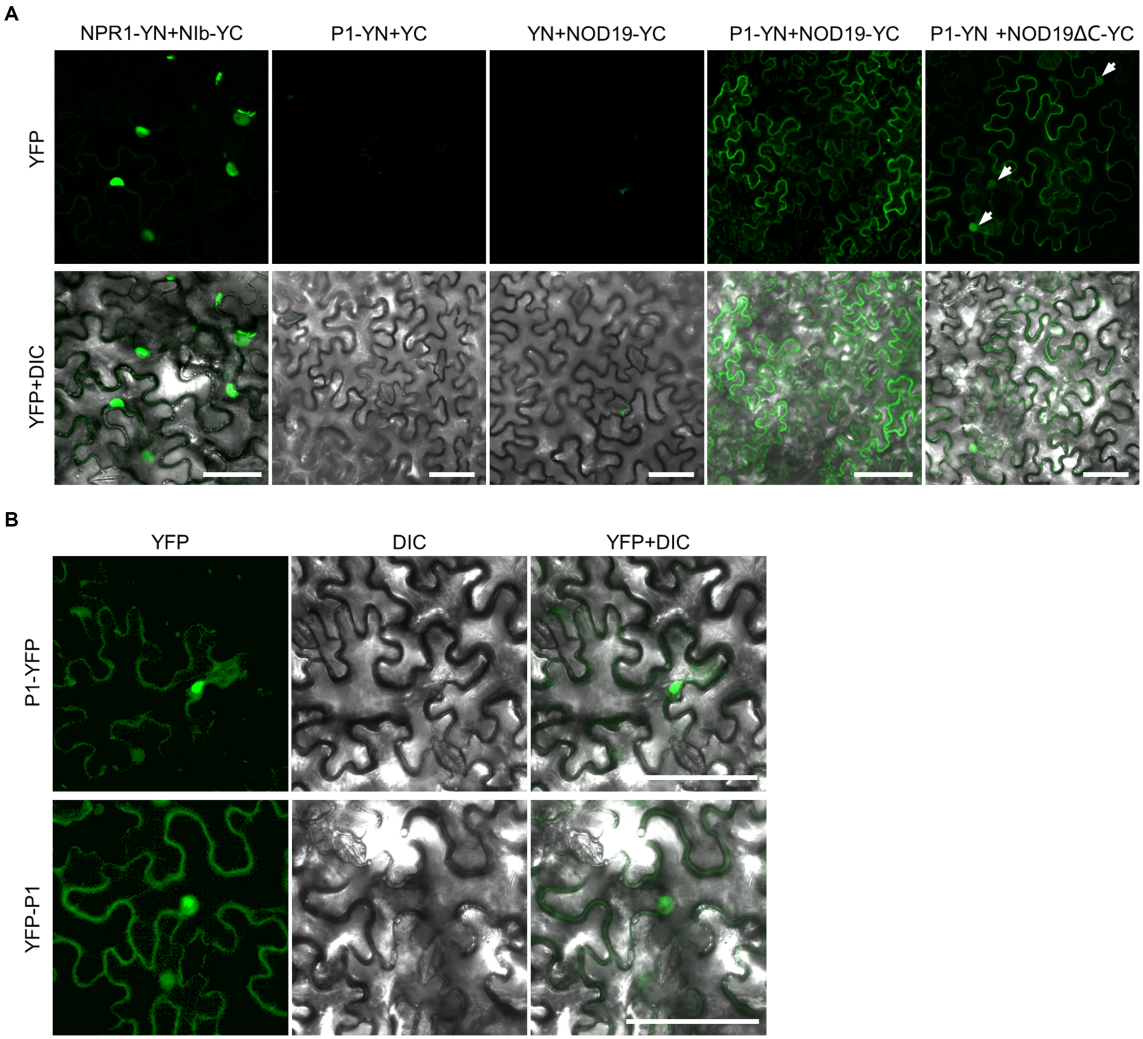
P1 interacts with NOD19. **(A)** Confocal microscope photos of epidermal cells of *N. benthamiana* leaves infiltrated with Agrobacterium harboring YN and YC, NPR1-YN and NIb-YC, P1-YN and NOD19-YC, or P1-YN and NOD19ΔC-YC. The nuclei are indicated by white arrows. All photos were taken at 2 dpi with identical parameters. **(B)** Confocal microscope photos of *N. benthamiana* epidermal cells expressing P1-YFP or YFP-P1 at 2 dpi. DIC, differential interference contrast channel. Scale bar = 50 μm.

Since only the N-terminal part of NOD19 was found in the three plasmids obtained from the Y2H screen, the full-length coding sequence of *NOD19* was cloned from *A. thaliana* and was used to confirm the interaction with P1. Unexpectedly, full-length Arabidopsis NOD19 failed to interact with TuMV P1 in the Y2H assay, suggesting that the missing C-terminal part of NOD19 may influence its interaction with TuMV P1. As an alternative, bimolecular fluorescence complementation (BiFC) assay was performed to confirm the interaction. Leaves of four-week-old *N. benthamiana* seedlings were infiltrated with *Agrobacterium* harboring N-terminal part of YFP (YN) and C-terminal part of YFP (YC)-tagged NOD19 (NOD19-YC), YC and C-terminal YN-tagged P1 (P1-YN), P1-YN and NOD19-YC, or NIb-YC and YN-tagged NON-EXPRESSOR OF PATHOGENESIS-RELATED GENES1 (NPR1-YN). At 2 days post-infiltration (dpi), the infiltrated leave areas were analyzed by a laser scanning confocal microscope. The results showed that the epidermal cells of *N. benthamiana* leaves infiltrated with *Agrobacterium* harboring NPR1-YN and NIb-YC, which were included as a positive control (Martínez et al., 2023), displayed bright fluorescence in the nucleus, while no fluorescence was observed in those infiltrated with Agrobacterium harboring P1-YN plus YC or YN plus NOD19-YC (Figure 2A), confirming the specificity of the BiFC assay in our experimental conditions. Epidermal cells of *N. benthamiana* leaves infiltrated with P1-YN and NOD19-YC also showed bright fluorescence (Figure 2A), indicating that TuMV P1 interacts with full-length NOD19. Moreover, in contrast to the nuclear fluorescence from the interaction between NIb and NPR1, the fluorescence from the interaction between P1 and NOD19 did not localize the nucleus, but instead at the cell periphery, e.g., cytoplasm, on a membrane within or bordering the cytoplasm, or even in the extracellular space (Figure 2A). We thus analyzed the subcellular localization of P1 as N- or C-terminal YFP tagged recombinant protein in *N. benthamiana* epidermal cells. To avoid the release of P1 from the P1-GFP fusion, the last three amino acids of the cleavage site (Val-His-Phe) was deleted. Consistent with previous report of tobacco etch virus (TEV)-encoded P1 (Martínez and Daròs, 2014), TuMV P1 also distributed both in the cytoplasm and nucleus (Figure 2B). We further confirmed the interaction between TuMV P1 and a NOD19 mutant lacking the C-terminal 43 residues (NOD19ΔC) by BiFC. Results showed that the fluorescence from the interaction between P1-YN and NOD19ΔC-YC was observed in both the nucleus and cytosol. Together, these data suggest that full-length Arabidopsis NOD19 may only interact with TuMV P1 outside the nucleus, which explains the negative results of the Y2H assay.

### 2.3. NOD19 is a membrane protein that expressed mainly on aerial parts of Arabidopsis

**Figure 3:**
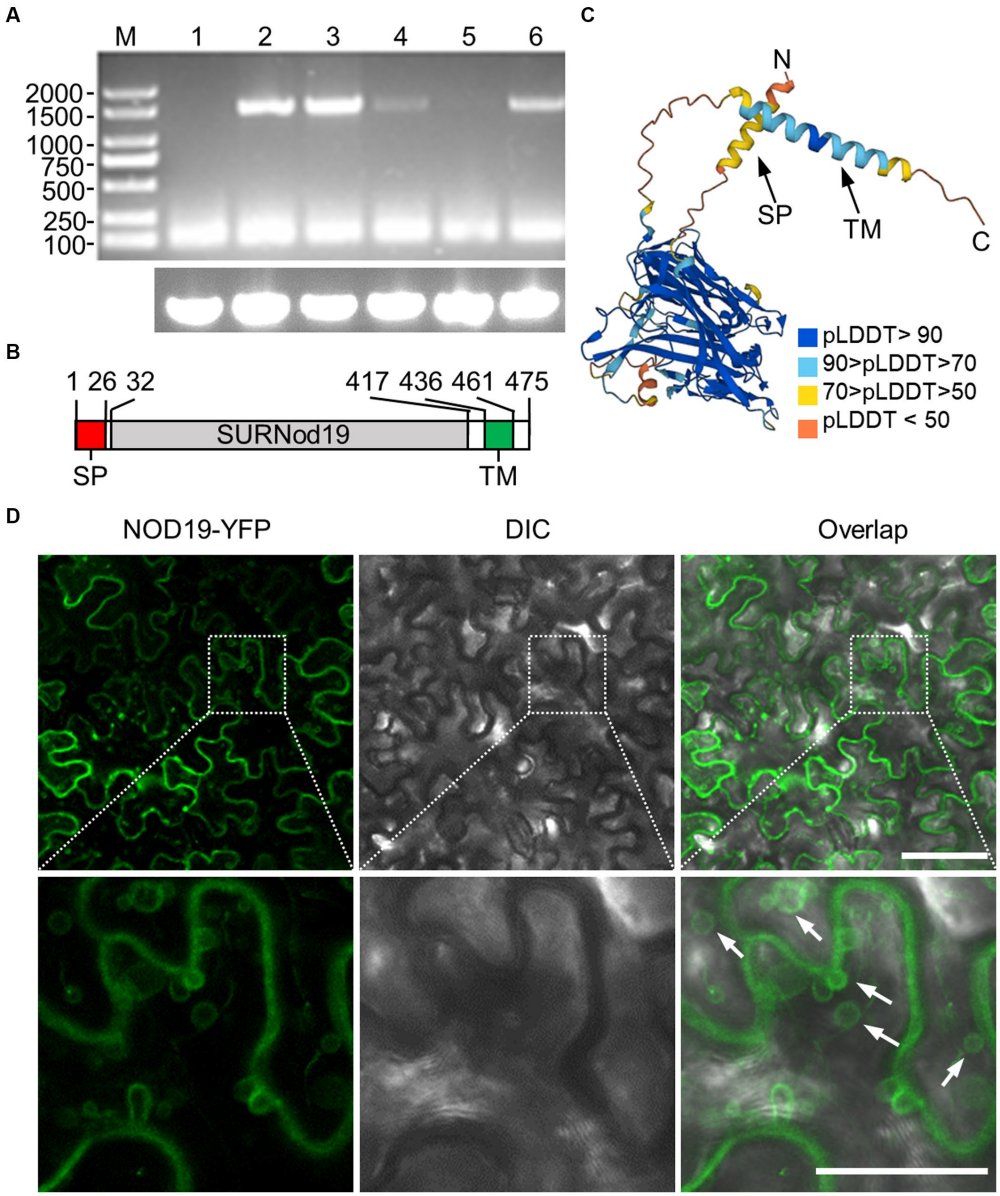
NOD19 is a membrane-associated protein. **(A)** Photograph of 1% agarose gel electrophoresis showing the RT-PCR products of the full-length transcript of *NOD19*. Lane M, DL2000 Plus DNA Marker; Lanes 1 to 6 representing RT-PCR results with cDNA from root, shoot meristem, flower, leaf, seed, and stem, respectively. Amplificons of *ACTIN II* are showing at the bottom panel. **(B)** Schematic representation of NOD19. Numbers represent amino acid positions of domain boundaries. SP, signal peptide; TM, transmembrane motif; SURNod19, stress upregulated Nod 19 super family domain. **(C)** Cartoon representation of predicted NOD19. The structure is colored based on confidence values (blue to orange represents high to low confidence). The signal peptide and transmembrane motif are also indicated. **(D)** Confocal microscope photos of *N. benthamiana* epidermal cells expressing NOD19-YFP at 2 dpi. The lower panel represents enlarged images of the dashed area in the top panel. White arrows indicating the typical NOD19-YFP-induced vesicles. Scale bar = 50 μm.

We then determined the expression profile of *NOD19* in *A. thaliana* by reverse transcription-polymerase chain reaction (RT-PCR). The results showed that transcript of *NOD19* was detected in shoot meristem, flower, leaf, and stem, but not in the root or seed (Figure 3A). Bioinformatic analyses showed that the N-terminal 26 amino acid of NOD19 may function as a signal peptide, the middle part is the stress upregulated Nod 19 super family domain (SURNod19), while its C-terminal contains a hydrophobic helix (Figure 3B). We also analyzed the alphafold2-predicted structure of NOD19 and found that the middle SURNod19 domain of NOD19 is folded into a compact structure with high confidence score (Figure 3C). The predicted signal peptide at the N-terminal end and the putative transmembrane motif at the C-terminus formed into an α-helix structure with relative lower confidence score (Figure 3C). We further analyzed the subcellular localization of NOD19 using C-terminal YFP-tagged construct (NOD19-YFP) in *N. benthamiana* epidermal cells. We found that the fluorescence of NOD19-YFP was located at the cell periphery (Figure 3D). Detailed inspection showed that hollow fluorescent vesicles was always found near the cell periphery (Figure 3D). Together, these data suggest that NOD19 maybe a membrane-associated protein that may interact with endomembrane via a hydrophobic α-helix at the C-terminus.

### 2.4. Knockout NOD19 affects TuMV proliferation

**Figure 4:**
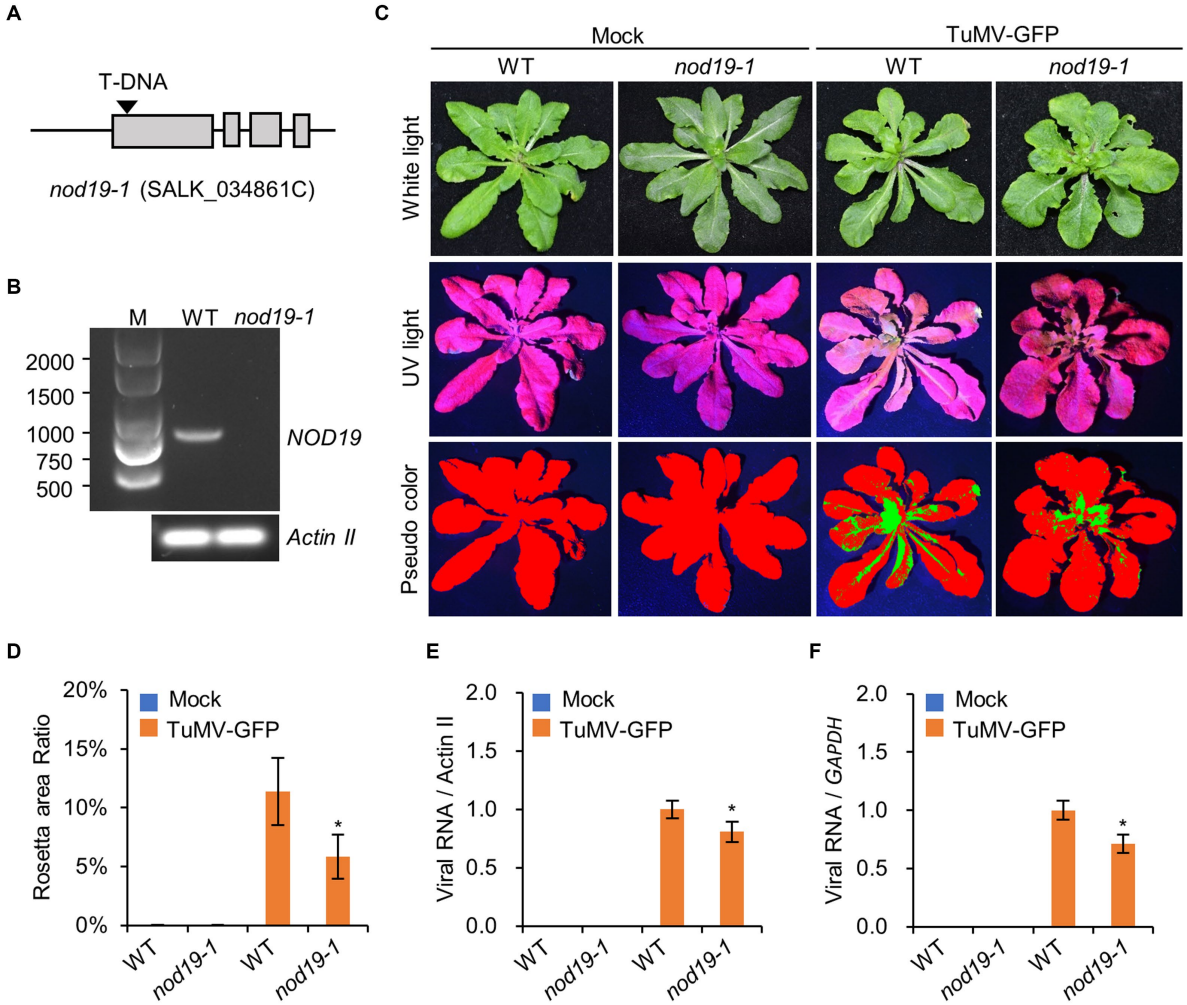
Knockout NOD19 affects viral proliferation. **(A)** Schematic representation of the insertion position of T-DNA on the *NOD19* gene in the *nod19-1* mutant. **(B)** Photograph of 1% agarose gel electrophoresis showing the PCR products of the full-length *NOD19* transcript on the template of cDNA from WT or *nod19-1* seedlings. Amplicons of *ACTIN II* are showing at the bottom panel. **(C)** Phenotypes of WT plants and *nod19-1* seedlings agroinfiltrated with infiltration buffer (mock) or Agrobacterium harboring TuMV-GFP under white light (WL) and ultraviolet light (UV) at 14 dpi. Red and green colors in the pseudocolor (PC) panel refer to virus-free and virus-infected areas, respectively. **(D)** Bar chart showing the ratios of TuMV-infected to the total leaf area of WT plants and *nod19-1* at 14 dpi. The bars represent the mean ± SD (*n* = 5). **(E,F)** Bar chart of the relative levels of viral RNA in WT plants and *nod19-1* at 14 dpi. RT–qPCR was performed with *ACTIN II* **(E)** or *GAPDH* **(F)** as the reference gene, and the viral RNA in the WT was taken as 1. The bars represent the mean ± SD (*n* = 5). *, indicates *p* < 0.01 in the Student’s *T*-test.

To understand the role of NOD19 in viral proliferation, we obtained a T-DNA insertion mutant of *NOD19* (*nod19-1*; SALK_034861C) from the Arabidopsis Biological Resource Center (ABRC). Genotyping, Sanger sequencing, and RT-PCR showed that the T-DNA was inserted in the first exon of *NOD19*, which caused the loss of full-length *NOD19* transcript (Figures 4A,B). Three-week-old seedlings of wild-type *A. thaliana* ecotype Col-0 (WT) or *nod19-1* were inoculated with TuMV-GFP, an infectious cDNA clone expressing a free GFP between P1 and HcPro cistrons (Cotton et al., 2009), by agroinfiltration. At 14 dpi, we found that about 11.4% leaf area of WT seedlings showed GFP signal from TuMV-GFP, while only about 5.8% leaf area of *nod19-1* seedlings showed GFP signal (Figures 4C,D). Statistical analyses showed that virus-infected leaf area on *nod19-1* was significantly lower than that on WT plants (Figure 4D). We further analyzed the amount of viral RNA in WT and *nod19-1* seedlings by amplifying a fragment of the *CP* gene through reverse transcription-quantitative PCR (RT–qPCR). Consistently, seedlings of *nod19-1* also accumulated less viral RNAs as compared with that in WT seedlings (Figures 4E,F), indicating that knockout NOD19 attenuates TuMV proliferation.

### 2.5. Knockdown the soybean homolog of NOD19 attenuates SMV proliferation

**Figure 5:**
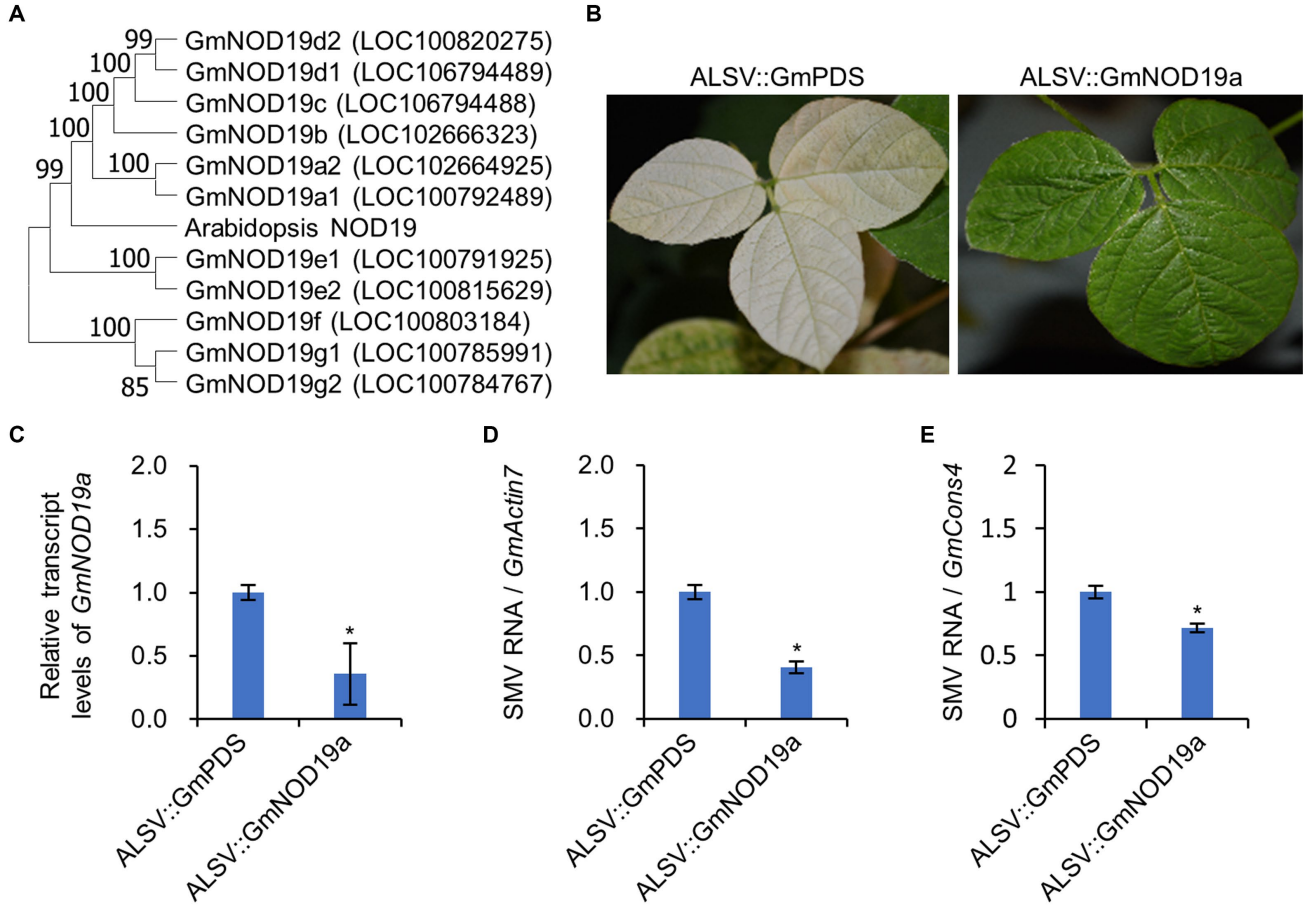
Knockdown soybean GmNOD19 attenuates SMV infection. **(A)** Phylogeny of soybean NOD19 paralogs. The phylogenetic tree was constructed using MEGA 11 with the neighbor-joining method. The numbers on the branches represent the percentage values of 1,000 bootstrap replicates. The gene locus numbers are indicated in brackets. **(B)** Phenotype of soybean Zhonghuang13 infected by ALSV::GmPDS or ALSV::GmNOD19a at 20 dpi. **(C)** Bar chart of the relative levels of *GmNOD19a* transcript. **(D,E)** Bar chart of the relative levels of SMV genome in soybean plants infected by ALSV::GmPDS and ALSV::GmNOD19a at 14 dpi. RT–qPCR was performed with the soybean *ACTIN 7* (*GmACT7*; **D**) or *CONSTITUTIVE 4* (*GmCONS4*; **E**) as the internal control, and the viral genome in the WT was normalized to 1. The bars represent the mean ± SD (*n* = 5). *, indicates *p* < 0.01 in the Student’s *T*-test. Experiment was repeated twice with similar trends.

We further analyzed the function of *NOD19* in the proliferation of other potyviruses on their respective hosts, i.e., SMV on its host soybean [*Glycine max* (L.) Merr.]. Soybean is an ancient tetraploid, which underwent two whole genome duplications. Most of soybean genes are paralogous genes with multiple copies. Blast search using the amino acid sequence of NOD19 as the query showed that there are at least 11 genes in *G. max* var. Willioms 82 with the of SURNod19-like domain in the genome of soybean. Phylogenetic analyses showed that six of the 11 soybean homologs were clustered with NOD19 of Arabidopsis in same cluster with *GmNOD19a1* and *GmNOD19a2* as the closest ones (Figure 5A). Multiple sequence alignment showed that the nucleotide sequences of *GmNOD19a1* and *GmNOD19a2* are also highly similar (Supplementary Figure 1). Thus, we tried to simultaneously silence *GmNOD19a1* and *GmNOD19a2* in soybean cultivar Zhonghuang13 using an ALSV-based RNA silencing vector. At 20 dpi, soybean plants infected by ALSV inserted with the fragment of soybean *PHYTOENE DESATURASE* (*GmPDS*) gene (ALSV::*GmPDS*) showed the photobleaching phenotype, while those infected by ALSV harboring *GmNOD19a* fragment (ALSV::GmNOD19a) displayed no obvious symptoms (Figure 5B). RT–qPCR showed that the expression level of *GmNOD19a* was reduced more than 60% in the systemic leaves infected by ALSV::GmNOD19a as compared with that in soybean plants infected by ALSV::GmPDS (Figure 5C). GmNOD19a-silenced soybean leaves were then mechanically inoculated by SMV strain SC7. At 2 dpi, the accumulation of SMV genomic RNA on the inoculated leaves were evaluated by amplifying a fragment of the *CP* gene of SMV through RT–qPCR. The results showed that seedlings inoculated by ALSV::GmNOD19a accumulated significantly fewer viral genome RNA than that on WT seedlings (Figures 5D,E), indicating that SMV infection is negatively influenced by *GmNOD19a* knockout.

## 3. Discussion

Despite the important role of P1 in the proliferation of potyviruses, only a few host factors have been identified thus far. In this study, a comprehensive Y2H screen was performed and eight host factors were successfully identified as potential P1-interacting host factors, indicating that Y2H is a powerful method for identifying P1-interacting host factors. The interaction between P1 and one candidate, namely NOD19, was analyzed in detailed. Previous studies suggest that *NOD19* responded to abiotic stress such as cold and copper toxicity (Naya et al., 2014; Mori et al., 2018). NOD19 is also targeted by microRNA398 (Naya et al., 2014). However, the specific function of NOD19 is still elusive at present. In this study, our data showed that NOD19 contains a signal peptide on the N-terminus and a hydrophobic α-helix at the C-terminus, indicating that NOD19 may be a membrane protein. Indeed, P1 interacts with NOD19 outside the nucleus, YFP-tagged NOD19 located exclusively at the cell periphery of epidermal cells of *N. benthamiana* and able to form vesicles. Together, these data clearly showed that NOD19 is a membrane-associated protein that may be exported into vacuole or other membranous organelles or secreted into the extracellular spaces. Viral infectivity studies showed that either knockout of NOD19 in Arabidopsis or knockdown the homolog of Arabidopsis NOD19 in soybean attenuates the infection of two potyviruses indicating that *NOD19* is either required for potyviral infection or is a negative regulator of plant antiviral immunity. We are trying to investigate these possibilities at present.

Many viruses adopt the synthesis and subsequent cleavage of precursor polyproteins to regulate protein expression. The proteinase that cleaves the polyprotein of viruses in the family *Flaviviridae* requires DNAJC14, a heat shock protein 40 (Hsp40) cochaperone (Yi et al., 2011; Bozzacco et al., 2016; Isken et al., 2019). Silencing of DNAJC14 results in impaired replication, which makes it an ideal antiviral target (Yi et al., 2011). It has already known that the cleavage of P1 from potyviral polyprotein by the C-terminal serine protease domain also need an unknown host factor. Thus, identification of the host factor is the key to understand potyviral infection process and development of novel antiviral strategy. Knockout NOD19 does not prevent the infection of TuMV-GFP (Figure 4), thus NOD19 is not the host factor required for the proteolytic activity. Several Y2H screens including this one have been performed to identify co-factor of P1 protease of SMV or TuMV (Shi et al., 2007; Pan, 2016). Moreover, a biochemical approach that combines *in vitro* translation assays and mass spectrometry analysis was used to identify P1 cofactor (Shan, 2018). However, all methods have been proven to be failed in identifying the proteinase cofactor. Thus, it is possible that P1 only requires the cofactor transiently and these methods are not suitable for identifying such host factor. Novel and more sensitive methods, e.g., proximity labeling (Mair et al., 2019), are needed to identify this transient protein–protein interactions.

Among the eight P1-interacting host factors, several ones have been reported to be involved in plant responses to biotic stress or plant-virus interaction. For instance, NTF2 is a Ras-GAP SH3-domain-binding protein (AtG3BP), which localizes to plant stress granules and has been shown to be involved in the infection of several unrelated viruses, i.e., pea necrotic yellow dwarf virus, abutilon mosaic virus, and pea enation mosaic virus 2 (Krapp et al., 2017; Brown et al., 2021). TCP21 is a transcriptional repressor that was recently found involved in the regulation of several plant hormones including the jasmonic acid (JA; Chen et al., 2023). Interestingly, P1 of TuMV also suppresses JA biosynthesis (Ji et al., 2021). Thus, it is also possible that P1 suppresses JA signaling also by interacting with TCP21 together by degrading cpSRP54 to block the delivers of ALLENE OXIDE CYCLASES (AOCs), key JA biosynthesis enzymes, onto the thylakoid membrane (Ji et al., 2021). We are trying to dissect the biological function of these P1-interacting host factors at present. In conclusion, eight P1-interacting host factors were identified by Y2H screen. Among which, the interaction between P1 and NOD19 was analyzed and found it was required for robust viral infection.

## 4. Materials and methods

### 4.1. Plant materials, virus inoculation, and sampling

*A. thaliana* ecotype Col-0 and *N. benthamiana* seeds were maintained by our lab and Soybean (*G. max*) cultivar Zhonghuang-13 seeds were kindly provided by the Heilongjiang Academy of Agricultural Science. Seedlings of *A. thaliana* and *N. benthamiana* were grown in a plant growth chamber under a 16-/8-h photoperiod at 23°С. Soybean seedlings were grown under a 16-/8-h photoperiod at 26°С in a greenhouse. Agroinfiltration was performed as described earlier with seedlings of the same age and similar sizes (Wu et al., 2022). In brief, *Agrobacterium* strain GV3101 harboring the proper plasmid were washed with infiltration buffer (10 mM MES, pH 5.6, 10 mM MgCl; 100 μM acetosyringone) twice by low-speed centrifuge at room temperature and infiltrated into *N. benthamiana* leaves at the indicated OD values. Mechanical inoculation was performed as described earlier (Li et al., 2022). The whole aerial part was taken for further analyses, e.g., RNA extraction and Western blot.

### 4.2. Plasmid construction

Full-length coding sequences of turnip mosaic virus (TuMV) *P1* and Arabidopsis *NOD19* (AT5G61820) were amplified using the primers listed in Supplementary Table 1 with the Phanta DNA polymerase (Vazyme, Nanjing, China) and inserted into a modified pDONR207m vector by a ClonExpress II One Step Cloning Kit (Vazyme). The gateway-compatible pEarleyGate101 and pEarleyGate104 plant binary expression vectors were used to produce C- or N-terminal YFP-tagged constructs (Earley et al., 2006). The pGADT7 and pGADT7 or gateway-compatible version pGBKT7-DEST and pGADT7-DEST were used to produce plasmids for the Y2H assay. The pEarleyGate201-YN, pEarleyGate202-YC, YC-pEarleyGate100 and YN-pEarleyGat100 were used to generate constructs for the bimolecular fluorescence complementation (BiFC) assay (Lu et al., 2010). All plasmids were verified by Sanger sequencing.

### 4.3. Construction of cDNA library and Y2H screen

The cDNA library of Arabidopsis mRNA in the pGADT7 vector was constructed as described earlier (Xu et al., 2020). Y2H assays were performed using the yeast strain Golden (Clontech, Beijing, China) as described previously (Cheng and Wang, 2017). All yeast selective media were purchased from Coolaber Technology (Beijing) Co., Ltd.

### 4.4. Confocal microscope observation

The fluorescence was visualized with a TCS SP8 LIGHTNING Confocal Microscope (Leica, Wetzlar, Germany) as described previously (Chai et al., 2020). Sequential mode was used when necessary.

### 4.5. Image quantification

Plant phenotype images were quantified by Fiji, a distribution of ImageJ for biologists and computer scientists (Schindelin et al., 2012). In brief, images were firstly split into red (chloroplast fluorescence), green (GFP fluorescence), and blue channels. The analyze particles function in Fiji was used to analyze the total leaf area using the red channel pixels, the virus-infected area using the green channel pixels with size 100-infinity and circularity 0.0–1.0. The ROI manager function was used to produce the pseudocolor image.

### 4.6. Knockdown soybean GmNOD19 by apple latent spherical virus

Apple latent spherical virus (ALSV)-based gene silencing was performed as described earlier (Don et al., 2022). In brief, a 309 base-pair cDNA fragment of *GmNOD19* was amplified from soybean cultivar Zhonghuang-13 and inserted into the pALSV2 vector to construct pALSV2-GmNOD19. The pALSV1 were co-infiltrated with pALSV2-GmNOD19 or pALSV2-GmPDS into *N. benthamiana* seedlings. At 20 days post infiltration (dpi), viral particles were purified from systemic leaves of *N. benthamiana* by PEG method. The purified viral particles in 0.01 mol/L phosphate buffered saline (pH 7.0) were directly used to inoculate the first-round true leaves of Zhonghuang-13 seedlings that were pre-dusted with carborundum. At about 16 dpi, the photobleaching phenotype was observed on soybean plants inoculated with pALSV1 and pALSV2-GmPDS. Reverse transcription and quantitative polymerase chain reaction (RT–qPCR) was then used to check the silencing efficiency of *GmNOD19* in the systemic leaves of soybean plants inoculated by pALSV1 and pALSV2-GmNOD19. The GmNOD19-silenced or photobleaching leaves were then mechanically inoculated by soybean mosaic virus (SMV) N1 isolate and the virus replication was evaluated by RT–qPCR at 2 dpi using primers listed in Supplementary Table 1.

### 4.7. RT-qPCR

Total RNA was extracted using the Eastep® Super Total RNA Extraction Kit (Promega, Beijing, China) and reverse transcribed into complementary DNA (cDNA) by a HiScript III 1st Strand cDNA Synthesis Kit with gDNA wiper (Vazyme) with Oligo (dT)_20_ and random hexamers. RT–qPCR was performed on a CFX Connect qPCR System in a 20 μL volume system containing 4 μL of 5 μM of each primer, 10-fold-diluted cDNA, and 10 μL 2 × AceQ® Universal SYBR qPCR Master Mix (Vazyme). Arabidopsis *ACTIN II* (AT3G18780) and *GLYCERALDEHYDE 3-PHOSPHATE DEHYDROGENASE* (GAPC2; At1g13440) and soybean *ACTIN 7* (*GmACT7*; LOC100806695) and *CONSTITUTIVE 4* (*GmCONS4*; LOC100783869) were used as the internal controls. All primers were listed in Supplementary Table 1.

### 4.8. Statistical analyses

Two-sided Student’s *T*-test was performed with Office Excel. The data are represented as the mean ± SD as indicated.

### 4.9. Bioinformatics analyses

DeepTMHMM (Hallgren et al., 2022), SignalP 6.0 (Teufel et al., 2022), and Phobius predictors (Käll et al., 2007) are used to infer signal peptides and transmembrane regions. Conserved domain was searched with the CDD/SPARCLE (Lu et al., 2020). Alphafold2-predicted structure of NOD19 was downloaded from the AlphaFold Protein Structure Database.^1^ The structure was visualized and rendered by iCn3D structure viewer.^2^

## Data availability statement

The original contributions presented in the study are included in the article/Supplementary material, further inquiries can be directed to the corresponding authors.

## Author contributions

X-YW and XC: conceptualization. EOB, YY, and YF: data acquirement. EOB, YY, YF, and MC: data analysis. X-XW, X-YW, and XC: supervision, funding acquisition, and review and editing. MC, XJ, and YL: methodology. XJ and YL: resources. YW and YG: validation. EOB: original draft. All authors contributed to the article and approved the submitted version.

## Funding

This research was funded by the National Natural Science Foundation of China (grant number 32022071) and the Natural Science Foundation of Heilongjiang Province, grant numbers (TD2022C003 and LH2019C027).

## Conflict of interest

The authors declare that the research was conducted in the absence of any commercial or financial relationships that could be construed as a potential conflict of interest.

## Publisher’s note

All claims expressed in this article are solely those of the authors and do not necessarily represent those of their affiliated organizations, or those of the publisher, the editors and the reviewers. Any product that may be evaluated in this article, or claim that may be made by its manufacturer, is not guaranteed or endorsed by the publisher.

## Footnotes

^1^www.alphafold.ebi.ac.uk

^2^https://www.ncbi.nlm.nih.gov/Structure/icn3d/icn3d.html
