# Supplementary material for: P1 of turnip mosaic virus interacts with NOD19 for vigorous infection
Source: Front Microbiol. 2023 Jun 23;14:1216950. doi: 10.3389/fmicb.2023.1216950 (PMC10326430; doi:10.3389/fmicb.2023.1216950)
Supplement: Supplementary file 1 [file Table_1.DOCX]

**Table S1** Primers used in the present study.

| **Primer name** | **Primer sequence (5′ to 3′)** |
| --- | --- |
| NOD19-LP | TTTATCCGATTCGTTCTCACG |
| NOD19-RP | ATCCAGGCCTTATAGCTCGAC |
| SALK_LBb1.3 | ATTTTGCCGATTTCGGAAC |
| pDONR207-P1-F | GTACAAAAAAGCAGGCTTCATGGCAGCAGTTACATTCGC |
| pDONR207-P1-R | CAAGAAAGCTGGGTCCCGACGAAAGTGCACAATCTTGTGAC |
| pDONR207-NOD19-F | GTACAAAAAAGCAGGCTTCATGATGGCTCGTTACCACAGG |
| pDONR207-NOD19-R | CAAGAAAGCTGGGTCCCGACGAGTACTAAGTGATTGGTAACC |
| TuMV-CP-rtF | CAGGTTTGACAGACGAGCAA |
| TuMV-CP-rtR | CCAGAGGTTCCAGCGTTTAC |
| AtActin II-rtF | CCGGTATTGTGCTGGATTCT |
| AtActin II-rtR | TTCTCGATGGAAGAGCTGGT |
| AtGAPC2-rtF | TCTCGATCTCAATTTCGCAAAA |
| AtGAPC2-rtR | CGAAACCGTTGATTCCGATTC |
| ALSV-NOD19-F | GTCAGGGCCCTGATTTCACATGCACAGAATGTAGATGTGAC |
| ALSV- NOD19-R | AATCTGGACCTTGTCCACCTAGAATGGTATATTCTGCCTGGCA |
| ALSV-RNA2-R | TGTGAAATCAGGGCCCTGACCTTCTAGCAGATTTGGGTC |
| ALSV-RNA2-F | GGTGGACAAGGTCCAGATTTTACTAAGATTATCTGGCCCACGGTC |
| SMV-CP-rtF | GGCAACGAGAACACAGTTCG |
| SMV-CP-rtR | ATCATCACCCACACGCCATT |
| GmNod19a-rtF | AGGGTTTTCATGGCCCAACGA |
| GmNod19a-rtR | AGCCATTTGTTCTCACACGATCA |
| GmCons4-rtF | GATCAGCAATTATGCACAACG |
| GmCons4-rtR | CCGCCACCACCATTCAGATTATGT |
| GmActin7-rtF | ATGAAGATTAAGGTCGTGGCAC |
| GmActin7-rtR | GTTTTTATCCGAGTTTGAAGAGGC |
