# Supplementary material for: P1 of turnip mosaic virus interacts with NOD19 for vigorous infection
Source: Front Microbiol. 2023 Jun 23;14:1216950. doi: 10.3389/fmicb.2023.1216950 (PMC10326430; doi:10.3389/fmicb.2023.1216950)
Supplement: Supplementary file 2 [file Presentation_1.PPTX]

## Slide 1
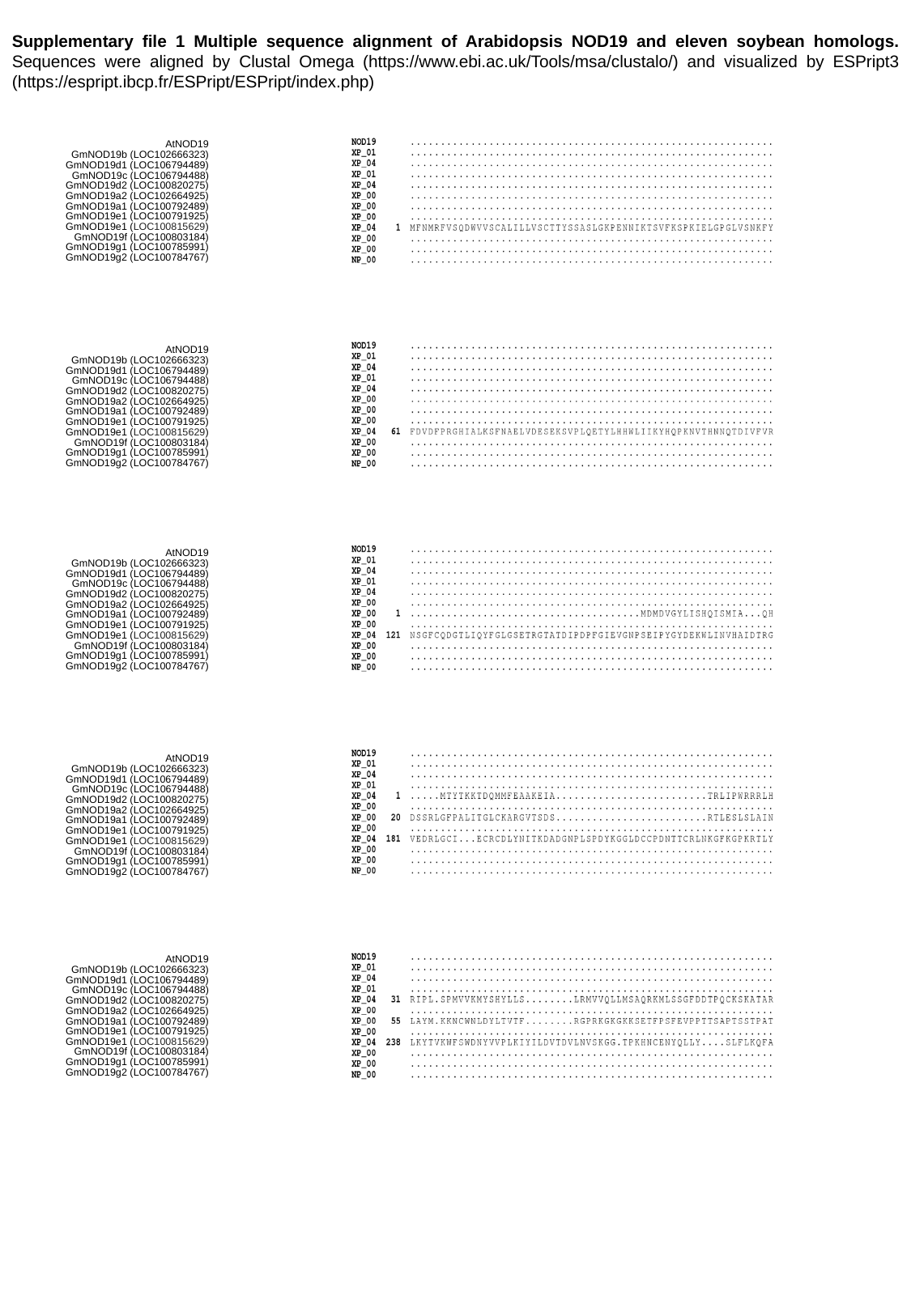

Supplementary file 1 Multiple sequence alignment of Arabidopsis NOD19 and eleven soybean homologs. Sequences were aligned by Clustal Omega (https://www.ebi.ac.uk/Tools/msa/clustalo/) and visualized by ESPript3 (https://espript.ibcp.fr/ESPript/ESPript/index.php)
AtNOD19
GmNOD19b (LOC102666323)
GmNOD19d1 (LOC106794489)
GmNOD19c (LOC106794488)
GmNOD19d2 (LOC100820275)
GmNOD19a2 (LOC102664925)
GmNOD19a1 (LOC100792489)
GmNOD19e1 (LOC100791925)
GmNOD19e1 (LOC100815629)
GmNOD19f (LOC100803184)
GmNOD19g1 (LOC100785991)
GmNOD19g2 (LOC100784767)
AtNOD19
GmNOD19b (LOC102666323)
GmNOD19d1 (LOC106794489)
GmNOD19c (LOC106794488)
GmNOD19d2 (LOC100820275)
GmNOD19a2 (LOC102664925)
GmNOD19a1 (LOC100792489)
GmNOD19e1 (LOC100791925)
GmNOD19e1 (LOC100815629)
GmNOD19f (LOC100803184)
GmNOD19g1 (LOC100785991)
GmNOD19g2 (LOC100784767)
AtNOD19
GmNOD19b (LOC102666323)
GmNOD19d1 (LOC106794489)
GmNOD19c (LOC106794488)
GmNOD19d2 (LOC100820275)
GmNOD19a2 (LOC102664925)
GmNOD19a1 (LOC100792489)
GmNOD19e1 (LOC100791925)
GmNOD19e1 (LOC100815629)
GmNOD19f (LOC100803184)
GmNOD19g1 (LOC100785991)
GmNOD19g2 (LOC100784767)
AtNOD19
GmNOD19b (LOC102666323)
GmNOD19d1 (LOC106794489)
GmNOD19c (LOC106794488)
GmNOD19d2 (LOC100820275)
GmNOD19a2 (LOC102664925)
GmNOD19a1 (LOC100792489)
GmNOD19e1 (LOC100791925)
GmNOD19e1 (LOC100815629)
GmNOD19f (LOC100803184)
GmNOD19g1 (LOC100785991)
GmNOD19g2 (LOC100784767)
AtNOD19
GmNOD19b (LOC102666323)
GmNOD19d1 (LOC106794489)
GmNOD19c (LOC106794488)
GmNOD19d2 (LOC100820275)
GmNOD19a2 (LOC102664925)
GmNOD19a1 (LOC100792489)
GmNOD19e1 (LOC100791925)
GmNOD19e1 (LOC100815629)
GmNOD19f (LOC100803184)
GmNOD19g1 (LOC100785991)
GmNOD19g2 (LOC100784767)

## Slide 2
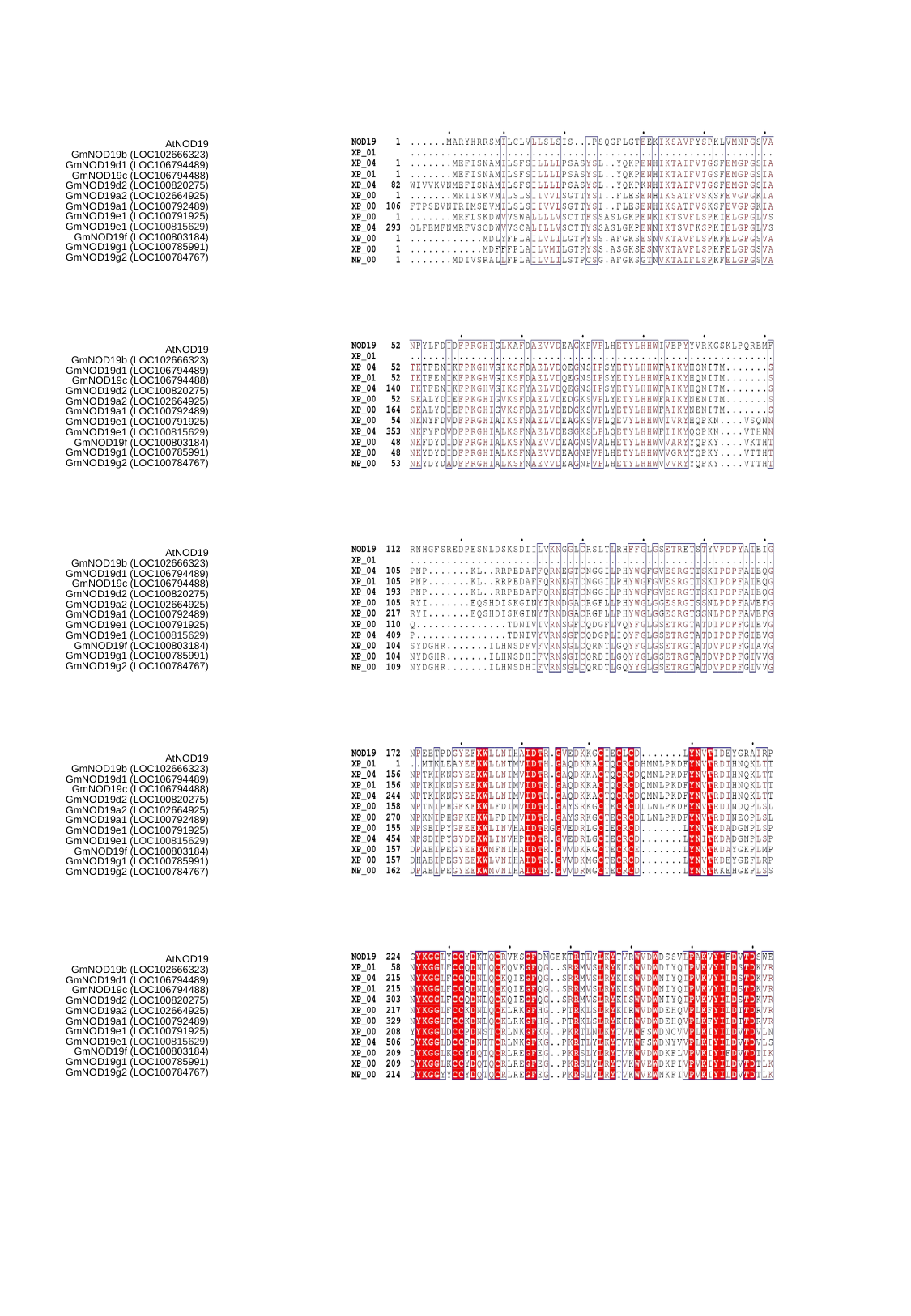

AtNOD19
GmNOD19b (LOC102666323)
GmNOD19d1 (LOC106794489)
GmNOD19c (LOC106794488)
GmNOD19d2 (LOC100820275)
GmNOD19a2 (LOC102664925)
GmNOD19a1 (LOC100792489)
GmNOD19e1 (LOC100791925)
GmNOD19e1 (LOC100815629)
GmNOD19f (LOC100803184)
GmNOD19g1 (LOC100785991)
GmNOD19g2 (LOC100784767)
AtNOD19
GmNOD19b (LOC102666323)
GmNOD19d1 (LOC106794489)
GmNOD19c (LOC106794488)
GmNOD19d2 (LOC100820275)
GmNOD19a2 (LOC102664925)
GmNOD19a1 (LOC100792489)
GmNOD19e1 (LOC100791925)
GmNOD19e1 (LOC100815629)
GmNOD19f (LOC100803184)
GmNOD19g1 (LOC100785991)
GmNOD19g2 (LOC100784767)
AtNOD19
GmNOD19b (LOC102666323)
GmNOD19d1 (LOC106794489)
GmNOD19c (LOC106794488)
GmNOD19d2 (LOC100820275)
GmNOD19a2 (LOC102664925)
GmNOD19a1 (LOC100792489)
GmNOD19e1 (LOC100791925)
GmNOD19e1 (LOC100815629)
GmNOD19f (LOC100803184)
GmNOD19g1 (LOC100785991)
GmNOD19g2 (LOC100784767)
AtNOD19
GmNOD19b (LOC102666323)
GmNOD19d1 (LOC106794489)
GmNOD19c (LOC106794488)
GmNOD19d2 (LOC100820275)
GmNOD19a2 (LOC102664925)
GmNOD19a1 (LOC100792489)
GmNOD19e1 (LOC100791925)
GmNOD19e1 (LOC100815629)
GmNOD19f (LOC100803184)
GmNOD19g1 (LOC100785991)
GmNOD19g2 (LOC100784767)
AtNOD19
GmNOD19b (LOC102666323)
GmNOD19d1 (LOC106794489)
GmNOD19c (LOC106794488)
GmNOD19d2 (LOC100820275)
GmNOD19a2 (LOC102664925)
GmNOD19a1 (LOC100792489)
GmNOD19e1 (LOC100791925)
GmNOD19e1 (LOC100815629)
GmNOD19f (LOC100803184)
GmNOD19g1 (LOC100785991)
GmNOD19g2 (LOC100784767)

## Slide 3
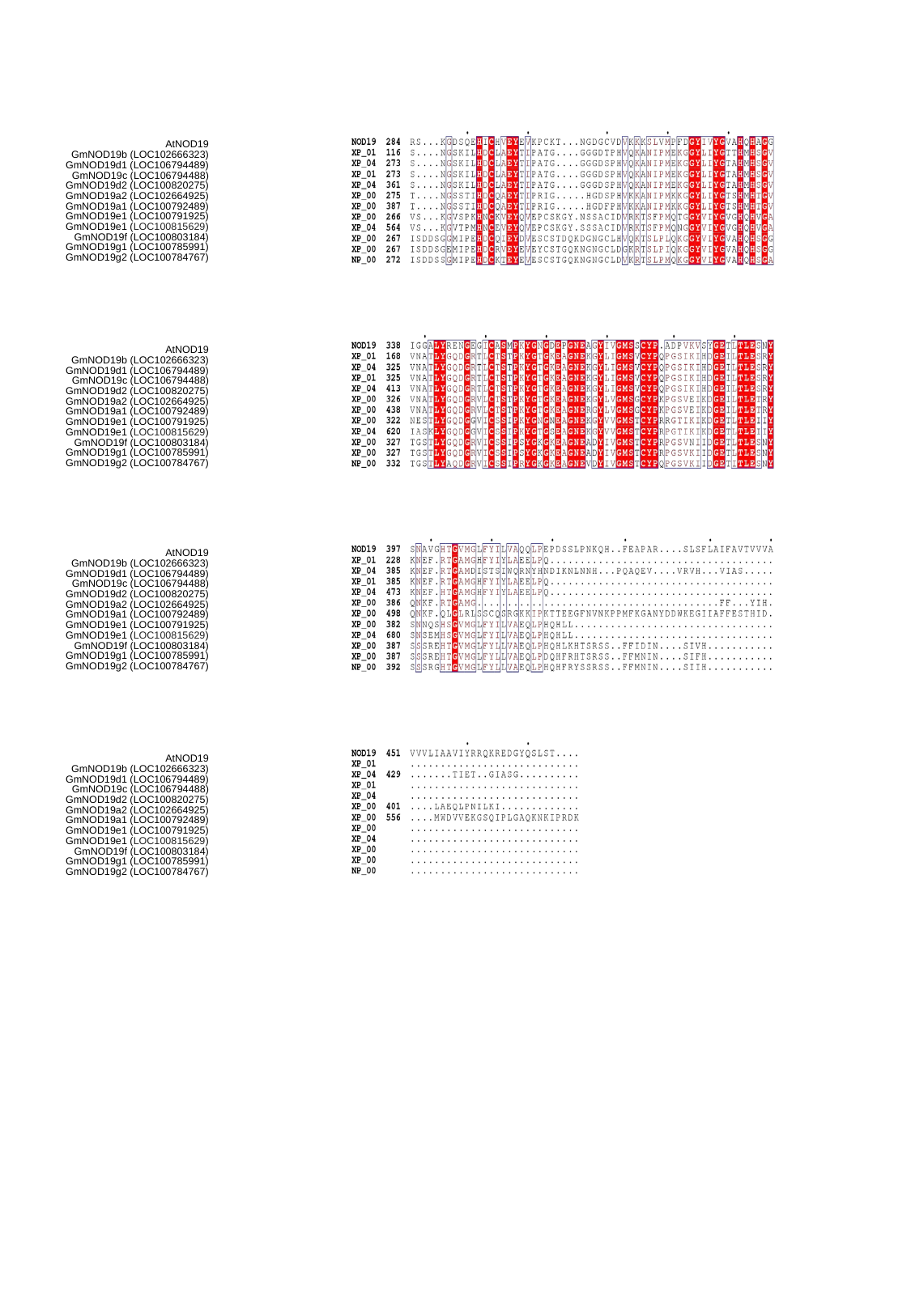

AtNOD19
GmNOD19b (LOC102666323)
GmNOD19d1 (LOC106794489)
GmNOD19c (LOC106794488)
GmNOD19d2 (LOC100820275)
GmNOD19a2 (LOC102664925)
GmNOD19a1 (LOC100792489)
GmNOD19e1 (LOC100791925)
GmNOD19e1 (LOC100815629)
GmNOD19f (LOC100803184)
GmNOD19g1 (LOC100785991)
GmNOD19g2 (LOC100784767)
AtNOD19
GmNOD19b (LOC102666323)
GmNOD19d1 (LOC106794489)
GmNOD19c (LOC106794488)
GmNOD19d2 (LOC100820275)
GmNOD19a2 (LOC102664925)
GmNOD19a1 (LOC100792489)
GmNOD19e1 (LOC100791925)
GmNOD19e1 (LOC100815629)
GmNOD19f (LOC100803184)
GmNOD19g1 (LOC100785991)
GmNOD19g2 (LOC100784767)
AtNOD19
GmNOD19b (LOC102666323)
GmNOD19d1 (LOC106794489)
GmNOD19c (LOC106794488)
GmNOD19d2 (LOC100820275)
GmNOD19a2 (LOC102664925)
GmNOD19a1 (LOC100792489)
GmNOD19e1 (LOC100791925)
GmNOD19e1 (LOC100815629)
GmNOD19f (LOC100803184)
GmNOD19g1 (LOC100785991)
GmNOD19g2 (LOC100784767)
AtNOD19
GmNOD19b (LOC102666323)
GmNOD19d1 (LOC106794489)
GmNOD19c (LOC106794488)
GmNOD19d2 (LOC100820275)
GmNOD19a2 (LOC102664925)
GmNOD19a1 (LOC100792489)
GmNOD19e1 (LOC100791925)
GmNOD19e1 (LOC100815629)
GmNOD19f (LOC100803184)
GmNOD19g1 (LOC100785991)
GmNOD19g2 (LOC100784767)
